# Elucidating the Degradation Pathways of Human Insulin in the Solid State

**DOI:** 10.1007/s41664-024-00302-5

**Published:** 2024-05-06

**Authors:** Andrew Fagan, Lorraine M. Bateman, Joseph P. O’Shea, Abina M. Crean

**Affiliations:** 1 SSPC, the SFI Centre for Pharmaceutical Research, School of Pharmacy, University College Cork, Cork, T12 YT20 Ireland; 2 School of Pharmacy, University College Cork, Cork, T12 YT20 Ireland; 3 Analytical and Biological Chemistry Research Facility (ABCRF), University College Cork, Cork, T12 YN60 Ireland; 4 School of Chemistry, University College Cork, Cork, T12 YN60 Ireland

**Keywords:** Insulin, Stability, Solid state, Deamidation, Covalent aggregation, Disulfide cleavage, Biopharmaceutical analysis

## Abstract

While there have been significant advances in the development of peptide oral dosage forms in recent years, highlighted by the clinical and commercial success of approved peptides such as Rybelsus®, there remain several barriers in the way of broad range applicability of this approach to peptide delivery. One such barrier includes the poor physical and chemical stability inherent to their structures, which persists in the solid state although degradation typically occurs at different rates and via different pathways in comparison to the solution state. Using insulin as a model peptide, this work sought to contribute to the development of analytical techniques for investigating common insulin degradation pathways. Chemically denatured, deamidated and aggregated samples were prepared and used to benchmark circular dichroism spectroscopy, reverse phase HPLC and size exclusion chromatography methods for the investigation of unfolding, chemical modifications and covalent aggregation of the insulin molecule respectively. Solid state degraded samples were prepared by heating insulin powder at 60 °C and 75% relative humidity for 1, 3, 5 and 7 d, and the degradation profiles of the samples were evaluated and compared with those observed in solution. While no unfolding was observed to occur, significant deamidation and covalent aggregation were detected. Reductive disulfide bond cleavage using dithiothreitol allowed for separation of the insulin A- and B-chains, offering a facile yet novel means of assessing the mechanisms of deamidation and covalent aggregation occurring in the solid state.

**Supplementary Information:**

The online version contains supplementary material available at 10.1007/s41664-024-00302-5.

## Introduction

Due to their remarkable efficacy, reduced systemic toxicity and high tolerability, proteins and peptides have emerged as highly effective therapies, particularly in the treatment of oncological and metabolic disorders [1, 2]. While the majority of peptide drugs on the market require delivery via subcutaneous injection, there has been increasing interest in alternative routes of administration, in particular the oral route [3–5]. This is highlighted by the recent FDA approval of oral formulations of octreotide (Mycapssa®), semaglutide (Rybelsus®) and desmopressin (Minirin®). Another interesting therapeutic peptide that would greatly benefit from an oral approach to delivery is insulin, which has received considerable attentions for advancing novel approaches to peptide delivery [6–9]. While much of the focus during oral peptide development has been on the improvement of intestinal absorption and oral bioavailability, another major hurdle in the way of capitalising on the many advantages of peptide therapeutics that must be addressed is their poor physical and chemical stability.

Poor stability of peptides during processing, storage and delivery into the body, can not only result in a decrease in overall therapeutic efficacy but is also associated with increased unwanted immunogenic side effects, such as disruption of normal protein–protein interactions and development of amyloid diseases [10–12]. There are two main categories of degradation that peptides may undergo: physical and chemical [13, 14]. Physical degradation refers to conformational changes related to secondary, tertiary and quaternary structures and includes unfolding and aggregation. Chemical degradation, on the other hand, refers to primary structural changes such as deamidation [15, 16]. The physical and chemical stabilities of peptides are intrinsically dependent on the properties of the molecule and indeed are connected to one another, i.e., molecular conformation affects susceptibility to chemical attack, while changes in primary structure likewise affect secondary, tertiary, and quaternary structures [17].

While the stability of insulin has been well characterised in solution, less work has been performed on the molecule in the solid state and the resultant implications for processing and manufacture of solid oral dosage forms. In solution, various external factors, such as agitation, temperature and freeze-thawing, along with the solution conditions, e.g., pH and ionic strength, significantly impact the stability of insulin. For example, the presence of air–liquid interfaces as a result of shaking is known to cause insulin to undergo aggregation and fibrillation, whilst chemical degradation in the form of deamidation can be initiated in acidic environments [18, 19]. In the solid state, on the other hand, the tightly fixed nature of the molecules restricts their mobility and, therefore, the type and extent of the degradation that can occur may be altered relative to the solution state. As such, chemical degradation pathways, which require only short-range conformational flexibility, predominate over physical degradation processes, which require much greater molecular mobility [20]. For example, insulin aggregation is observed to occur in the solid state, however, it is largely the result of intermolecular covalent interactions between insulin molecules and often requires elevated residual moisture content to increase conformational flexibility [21–24]. Additionally, in contrast to the solution state, temperature, humidity and light are the predominant factors affecting stability in the solid state, along with the solution conditions prior to lyophilisation [22, 23, 25–27].

Clearly a comprehensive understanding of the various degradation pathways of a peptide drug substance plays an important role in formulation design and development of solid oral dosage forms. To date, excipient choice has focused primarily on improving the oral bioavailability of peptides through the use of permeation enhancers and enzyme inhibition [28]. However, understanding the stability of the peptide drug substance to the drug product manufacturing processes and indeed to the long term storage conditions is essential to inform rational excipient screening and selection.

A large body of work has been performed in this field on the development and application of a multitude of complementary analytical methods for investigating the physical and chemical stability of human insulin [29]. This has highlighted the importance of deamidation and covalent aggregation in both the solution and solid state. However, while the mechanisms of deamidation have been well characterised, opposing covalent aggregation pathways have been observed in the solid state, including transamidation and disulfide exchange [22, 25]. As physicochemical characterisation of peptides is performed in the solution state, in this paper, the authors sought to build upon previous work by benchmarking commonly used analytical techniques with positive control samples that have followed known degradation pathways in the solution state. Circular dichroism (CD) spectroscopy and size exclusion chromatography (SEC) were chosen to investigate unfolding and aggregation respectively, while reverse phase high performance liquid chromatography (RP-HPLC) and mass spectrometry (MS) have been chosen to investigate chemical modifications. These techniques were then translated to the solid state to highlight their efficacy for elucidating the degradation pathways of samples exposed to high temperature and humidity conditions in the solid state, with the techniques offering an alternative approach to solid state characterisation to enable a platform screening approach. Reductive cleavage of disulfide bonds, using dithiothreitol as a reducing agent, was used as a simple but highly effective tool to confirm the mechanism of deamidation and covalent aggregation occurring in the solid state samples.

## Experimental

### Materials

Recombinant human insulin and all reagents used during analysis were procured from Merck KGaA, unless otherwise stated.

### Degraded Sample Preparation

Standard recombinant human insulin samples were reconstituted in 0.1% formic acid (FA), pH 2.6. Insulin samples were exposed to a range of conditions to induce degradation. Chemical denaturation was performed by incubating insulin samples at 1 mg/mL in 6 mol/L urea, prepared in 0.1% FA, at room temperature for 4 h [29, 30]. To initiate aggregation, 4 mg/mL samples, prepared in 0.1% FA, were heated at 60 °C for 18 h. Deamidated samples were prepared by storing insulin at 10 mg/mL in 0.1% FA for 1 month [31]. All samples were diluted to 1 mg/mL as required prior to analysis. All samples were prepared and measured in triplicate.

### Solid State Sample Preparation

Subsequently, 7 mg insulin samples were stored at 60 °C/75% relative humidity (RH) for 1, 3, 5 and 7 d to promote solid state degradation. Kilner® jars were used to store samples in a sealed environment, with constant RH conditions maintained using saturated sodium chloride solutions. The samples were placed in bespoke 3D printed Eppendorf holders (3D Jake) inside the Kilner® jars, and the stability chambers were placed in an oven at 60 °C. Prior to analysis, the solid state degraded samples were reconstituted in 0.1% FA to a target concentration of 1 mg/mL and centrifuged for 2 min at 14,000 r/min using a Mikro 120 Centrifuge (Hettich) to separate insoluble particles. All samples were prepared and measured in triplicate.

### Disulfide Bond Cleavage

Dithiothreitol (DTT) was used as a reducing agent to separate the insulin A- and B-chains via disulfide cleavage. DTT was prepared in 100 mmol/L phosphate buffer at pH 8.0 with 6 mol/L urea and added to samples at a final DTT concentration of 50 mmol/L. Urea was used to unfold the protein to enable exposure of the disulfide bonds to the surface of the protein and ensure complete reduction. All insulin standard and degraded samples were prepared in 50 mmol/L DTT solutions at a target concentration of 1 mg/mL. Where degradation was performed in the solution state, as in the case of the deamidated samples, the samples were diluted to a final insulin concentration of 1 mg/mL and final DTT concentration of 50 mmol/L. The samples were then heated on a ThermoMixer^TM^ Comfort (Eppendorf) at 65 °C for 30 min at 300 r/min. Samples were centrifuged for 2 min at 14,000 r/min using a Mikro 120 Centrifuge (Hettich) and analysed in triplicate.

### Circular Dichroism Spectroscopy

Circular dichroism (CD) spectroscopy was performed using a Chirascan^TM^ CD spectrometer (Applied Photophysics) fitted with an AWC 100 cooler (Julabo) and a TC 125 temperature controller (Quantum Northwest Inc.). The spectral measurement range used was 200–260 nm, with a bandwidth of 1 nm, a step-size of 1 nm and a time-per-point of 0.5 s. Each spectrum was recorded in triplicate, background subtracted and averaged. The spectrum of a blank sample was also recorded and subtracted from the averaged sample spectra. 0.01 cm pathlength Quartz Suprasil demountable cells (Hellma Analytics) were used. Data visualisation was performed using OriginPro 2022b graphing and analysis programme (OriginLab Corporation) and spectral smoothing was performed using the Sovitzky-Golay method, with a polynomial order of 2 and 8 points to the window.

### Size Exclusion Chromatography

An Agilent 1260 Series HPLC system with an AdvanceBio SEC 13 nm, 2.7 μm, 7.8 mm × 300 mm column (Agilent Technology) was used for the chromatographic separations. The mobile phase (MP) consisted of 65% L-arginine (1 g/L)/ 20% acetonitrile (ACN)/15% acetic acid. An isocratic elution method was used, with a total run time of 12 min required. A flow rate of 1 mL/min and injection volume of 5 μL were used. 280 nm was used as a protein specific detection wavelength. OriginPro 2022b was used for graphing chromatograms, and GraphPad Prism 8.4.3 was used for data analysis and graphical representation of results.

### Liquid Chromatography – Mass Spectrometry

For RP-HPLC, an Agilent 1260 Series HPLC system was used, while liquid chromatography – mass spectrometry (LC–MS) analysis was carried out using a Waters Acquity I-class UPLC coupled to a Vion IMS Mass Spectrometer (Waters Corporation), *m/z* range of 0–1250. All analysis was performed in positive ion mode. A Poroshell 120 SB-C18, 2.7 µm, 4.6 mm × 150 mm column (Agilent Technology) was used for chromatographic separations. MP A consisted of 95% H_2_O, 5% ACN and 0.1% formic acid (FA) and MP B consisted of 5% H_2_O, 95% can and 0.1% FA. The FA was used as a weak ion-pairing reagent for adequate adsorption of protein to the stationary phase, also adjusting the MP pH to approximately 2.6. A gradient elution method was used, where MP B was linearly increased from an initial content of 20% to 35% over 4 min, returned to 20% MP B in 1 min and held for 3 min. The flow rate was 1 mL/min and a total injection volume of 5 μL was used. The HPLC column was heated at 60 °C. 280 nm was used as a protein specific detection wavelength. Visualisation and data analysis were performed as in Sect. 2.5.

## Results and Discussion

In order to track the different degradation pathways that insulin may follow under stressed conditions, the first part of this investigation sought to establish the ability of several techniques to characterise changes to the insulin native structure. Insulin samples were subjected to chemical denaturants, heat and acidic conditions to stimulate degradation and compared subsequently with standard reference samples for identification of changes to the native insulin structure.

**Fig. 1:**
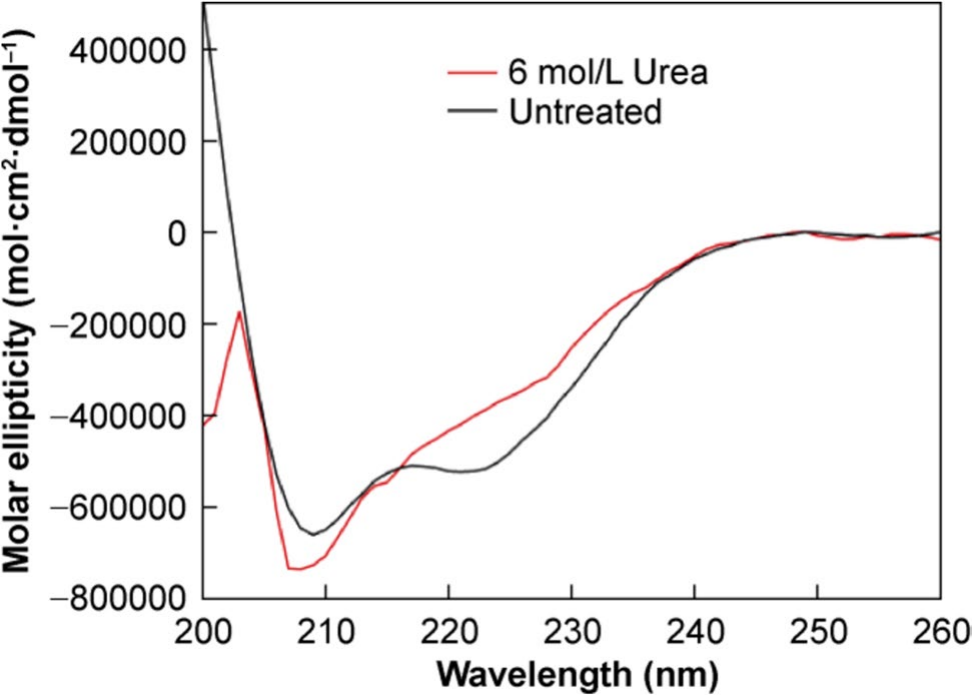
Representative far-UV CD spectra of a 1 mg/mL insulin sample in 6 mol/L urea (red curve) and an untreated 1 mg/mL insulin standard sample (black curve)

Firstly, far-UV CD spectroscopy was assessed for its ability to discriminate between insulin in its native fold and in unfolded states. A representative spectrum of a 1 mg/mL insulin standard sample is shown in the black curve in Fig. 1.

The presence of two negative bands at ~ 223 and 209 nm indicated that native insulin under these conditions was predominantly helical in nature, as expected [32]. Chemical denaturation was performed by incubation of insulin in 6 mol/L urea at room temperature for 4 h. Urea is known to cause a disruption of native hydrogen bonds along the polypeptide backbone, thus causing peptide and protein unfolding [29]. This can be clearly observed in the spectrum of a chemically degraded 1 mg/mL insulin sample shown in the red curve in Fig. 1. The unfolding was manifested by the loss of the negative band at 223 nm and the complete loss of spectral resolution below 200 nm (not shown). The negative band at 209 nm was present in the chemically degraded insulin spectrum, indicating that the insulin molecule was resistant to complete unfolding by high urea concentrations. The presence of the 209 nm band suggests some helical content was retained, shown previously to be the result of the formation of a partially unfolded monomeric species, known to precede aggregation and fibrillation [33].

The next degradation pathway investigated was aggregation and as such, SEC was used to separate native insulin from higher molecular weight species (HMWS) that may form during degradation. The compendial mobile phase, consisting of 65% L-arginine (L-Arg), 20% ACN and 15% acetic acid, was utilised in this investigation. Under these conditions, any non-covalent aggregates present in solution will dissociate and insulin exists exclusively as a monomer, while interactions with the stationary phase are minimised [34]. Therefore, any additional peaks present in the chromatogram are the result of covalent aggregation or fragmentation that occurred under stressed conditions.

**Fig. 2:**
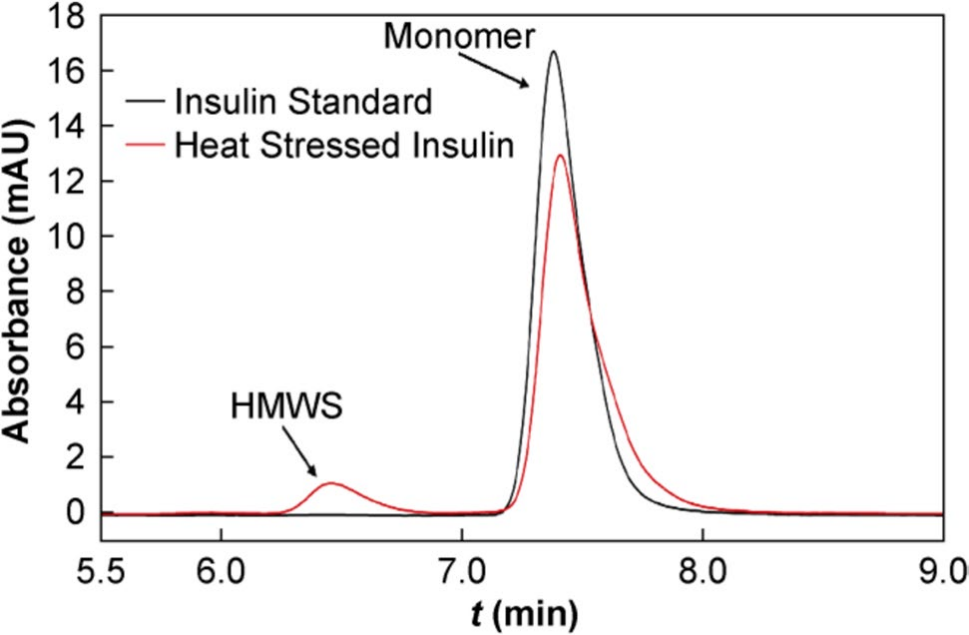
Representative SEC chromatogram of 1 mg/mL insulin standard sample (black curve) and 4 mg/mL insulin sample heated at 60 °C overnight and diluted to 1 mg/mL prior to analysis (red curve)

A representative chromatogram of a 1 mg/mL insulin standard sample is given in the black curve in Fig. 2. The insulin monomer appeared as a single peak in the spectrum and was monodisperse under these conditions. Degraded samples were also prepared by heating 4 mg/mL insulin at 60 °C overnight. The samples were diluted to 1 mg/mL and analysed using SEC. A representative chromatogram of a heat stressed sample is given in the red curve in Fig. 2. As expected, the insulin monomer was again observed to be present, however, an additional peak of lower intensity was found to elute approximately 1 min earlier than the monomer peak, indicating the presence of a HMWS in the sample. It is clear, therefore, that the stressed conditions caused insulin to undergo covalent aggregation, where the HMWS present were likely covalent insulin dimers (CIDs) [35].

Finally, LC–MS was investigated for its ability to separate and identify primary structural modifications of the insulin molecule. A particularly important chemical degradation pathway for insulin is deamidation. To promote deamidation, 10 mg/mL insulin samples were prepared in 0.1% FA and stored at room temperature for 1 month [31]. The deamidated samples were diluted to 1 mg/mL prior to analysis with LC–MS.

**Fig. 3:**
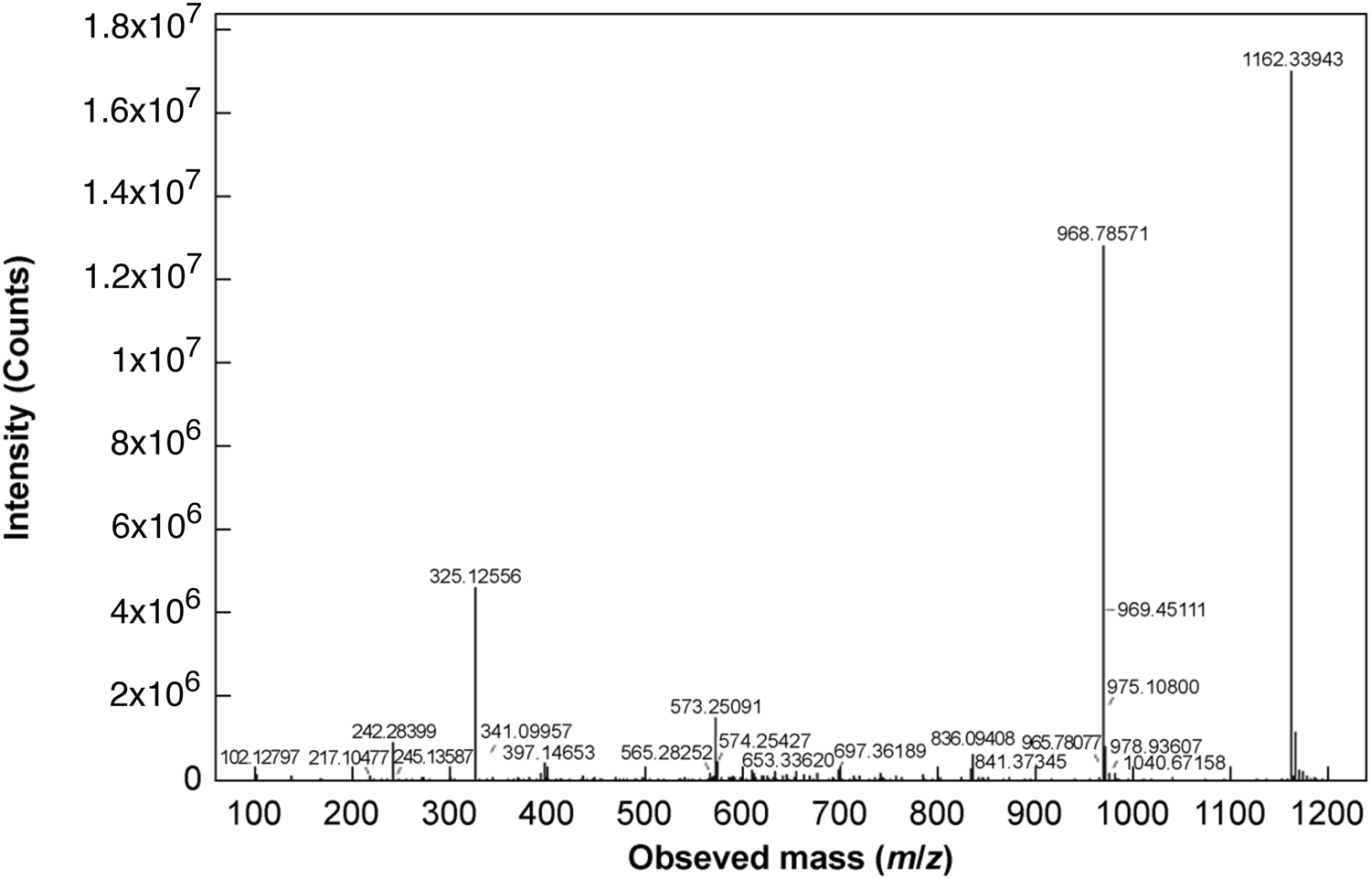
Representative mass spectrum of 1 mg/mL insulin standard sample in the *m*/*z* range of 0–1250. MS analysis was conducted in positive ion mode

**Fig. 4:**
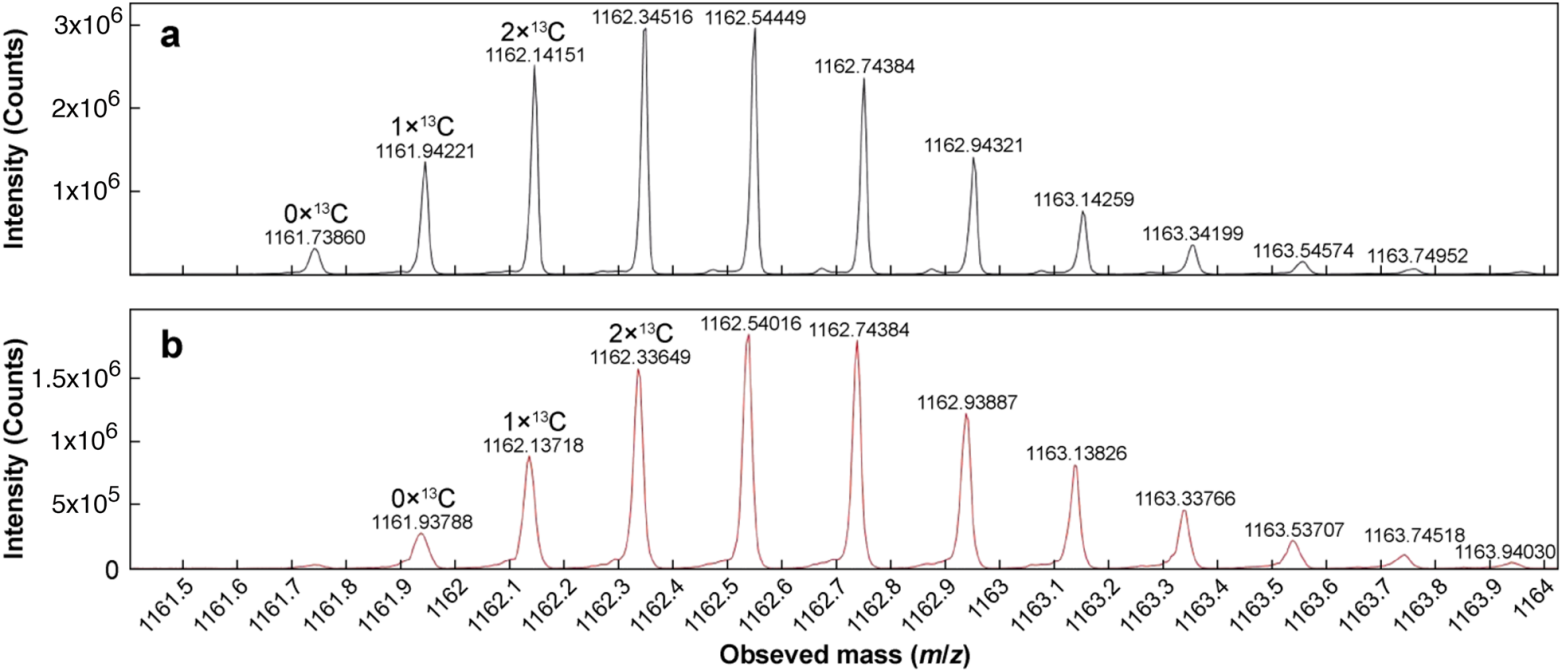
Peak clusters for the + 5 charge states from representative mass spectra of **a** native insulin and **b** degraded species. The monoisotopic peak and the ^13^C contributions for the first two isotopic peaks are indicated. MS analysis was conducted in positive ion mode

A representative mass spectrum of a 1 mg/mL standard insulin sample is given in Fig. 3. The predominant ions present appeared at 968.79 and 1162.34 *m/z*. Given the low pH of the solution (pH ~ 2.6), it is likely that these peaks represent insulin in the + 6 and + 5 charge states, respectively. Two unique species were observed to elute in the RP chromatograms of the deamidated samples (Fig. S1 in the supplementary information). These were separated on the reverse phase column, and the + 5 charge ions of the native insulin and the deamidated species are given in Fig. 4a and b, respectively.

From Fig. 4a, the monoisotopic peak of the + 5 charge state peak cluster for the native insulin was found to appear at 1161.74 *m/z*, while the peak cluster was centred at 1162.35 *m/z*, similar to that seen previously with the insulin standard sample in Fig. 3. However, in the mass spectrum of the degraded species, seen in Fig. 4b, a clear shift in the positions of all the ions can be seen, with the monoisotopic peak appearing at 1161.94 *m/z*, while the centre of the peak cluster shifted to 1162.54 *m/z*. This corresponded to an increase in 0.2 *m/z* for the + 5 charge state, and an overall increase in mass of 1 Da for the degradant species relative to the native insulin. Given that deamidation causes hydrolysis of an amide group to a carboxyl group, an increase in mass by 1 Da was expected. This increase served as confirmation that the new species observed was indeed the product of deamidation [36]. Analysis of the + 6 charge states also indicated an increase in mass of 1 Da, as shown in Table S1 in the supplementary information.

**Fig. 5:**
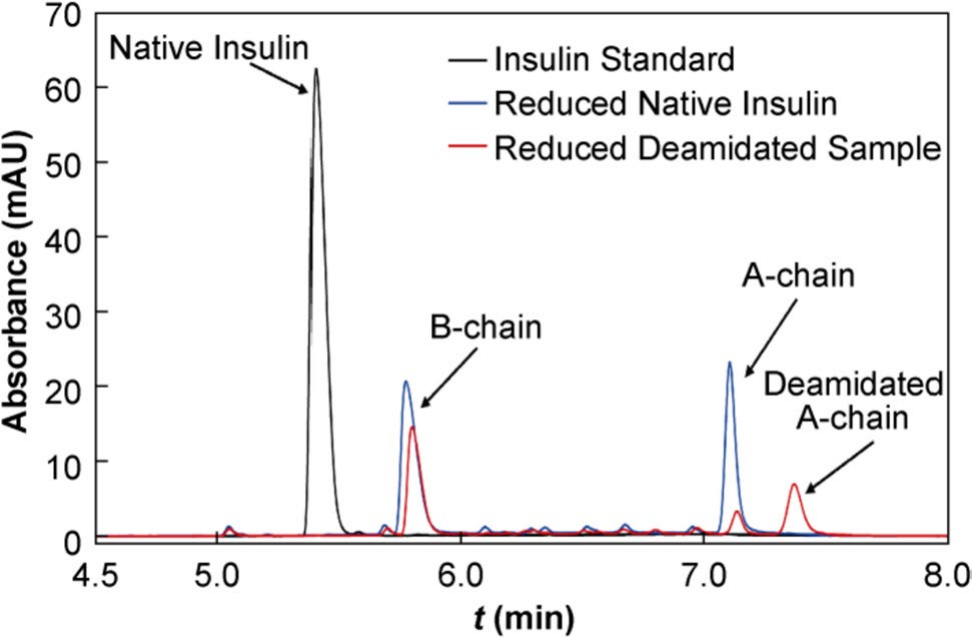
Representative RP-HPLC chromatograms of a 1 mg/mL insulin standard sample (black curve) and native (blue curve) and deamidated (red curve) insulin samples in 50 mmol/L DTT and 6 mol/L urea, heated at 65 °C and stirred at 300 r/min for 30 min

To identify the site of deamidation, insulin was separated into its constituent A- and B-chains using DTT. DTT is a dithiol reducing agent commonly used to reversibly reduce disulfide bonds in proteins [37]. To ensure complete reduction, DTT was used in large excess and combined with 6 mol/L urea to unfold the insulin molecule and expose all disulfide bonds to the surface. Representative chromatograms of standard and deamidated insulin samples reduced with 50 mmol/L DTT are given in Fig. 5.

**Fig. 6:**
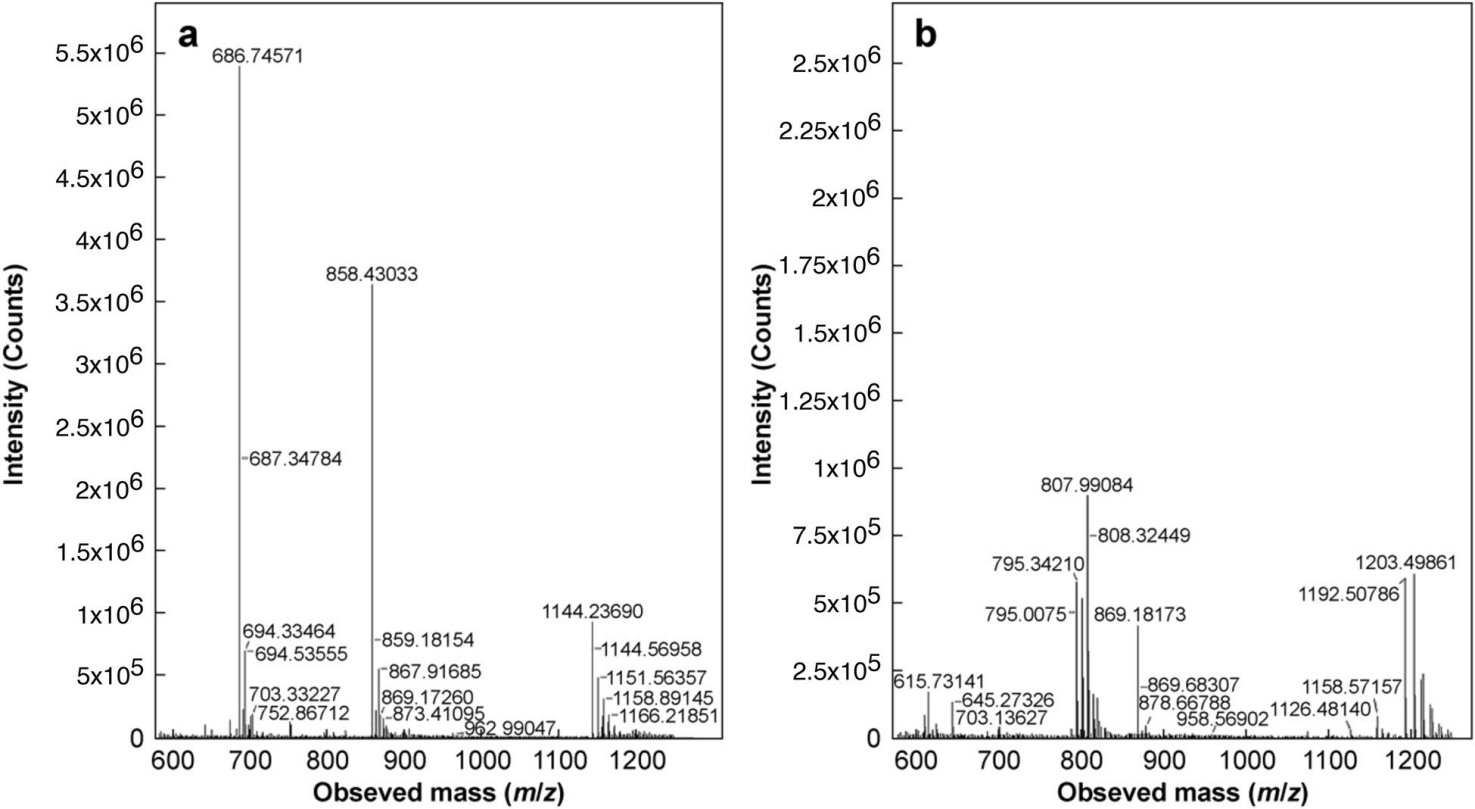
Representative mass spectra of **a** the peak at 5.4 min and **b** the peak at 7.1 min in the RP chromatograms over a mass range of 600–1250 *m/z* for a native insulin sample treated with 50 mmol/L DTT and 6 mol/L urea heated at 65 °C and stirred at 300 r/min for 30 min. MS analysis was conducted in positive ion mode

A single peak was observed to elute at 5.4 min in the chromatogram of a standard, untreated insulin sample, representing the native insulin molecule, as seen in Fig. 5. However, complete loss of the peak at 5.4 min occurred on treatment of a standard insulin sample with 50 mmol/L DTT, while 2 new species eluted at 5.8 min and 7.1 min, respectively. The loss of the native insulin peak and appearance of two unique peaks indicated successful cleavage of the disulfide bridges and presence of the separated A- and B-chains in solution. The identities of the peaks were elucidated with MS analysis, where representative mass spectra of the peaks at 5.4 min and 7.1 min are given in Fig. 6a and b, respectively. The predominant ions present in the mass spectrum of the peak at 5.4 min appeared at 1144.24 *m/z*, 858.43 *m/z* and 686.75 *m/z*. As the B-chain has a molecular weight of approxly 3400 Da, these ions likely represent the + 3, + 4 and + 5 charge states of the B-chain. As seen from Fig. 6b, multiple charging was evident from the presence of ions of low intensity at 1203.5 *m/z* and 807.99 *m/z*, suggesting the existence of the A-chain in + 2 and + 3 charge states.

**Fig. 7:**
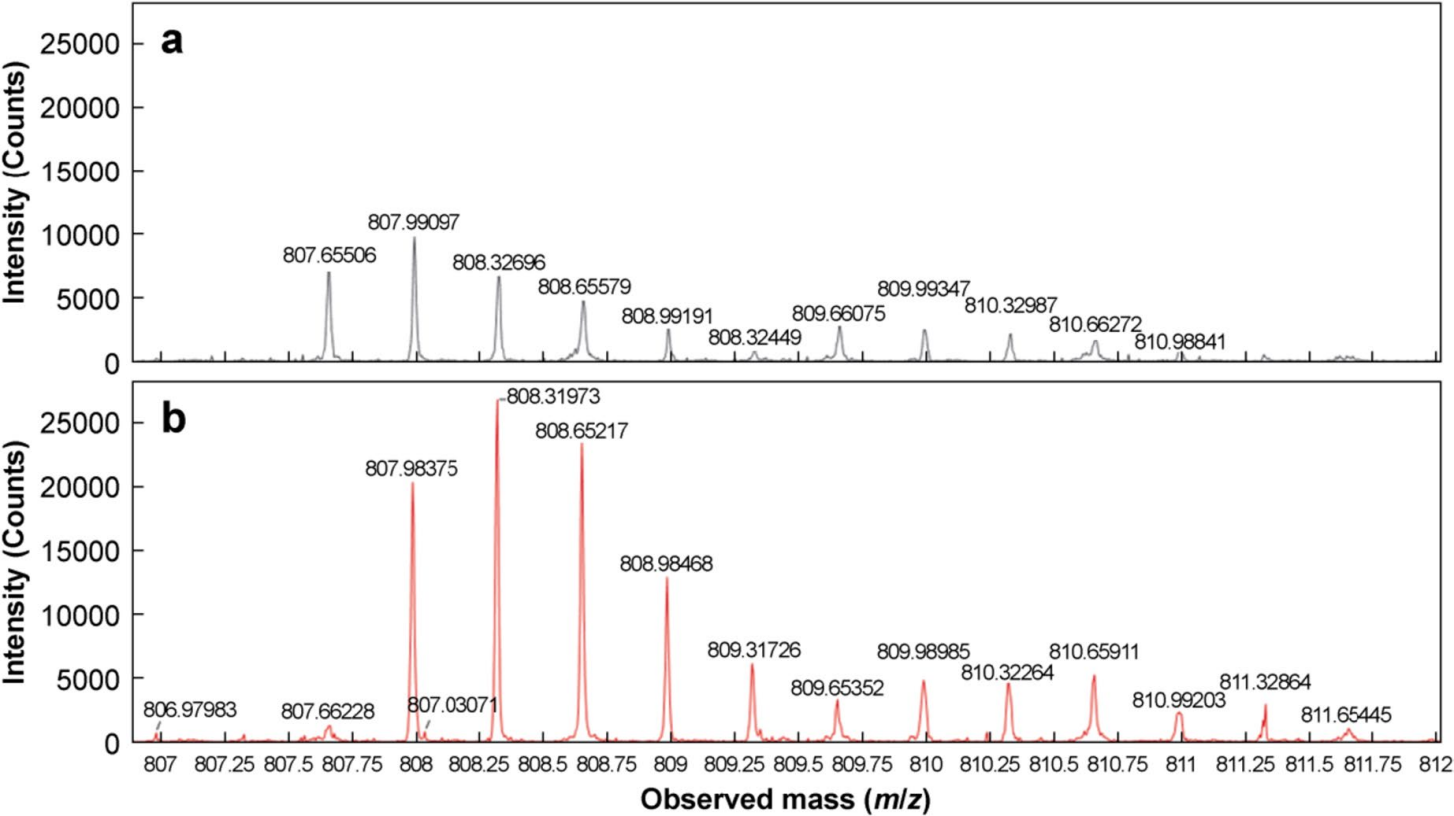
Peak clusters for the + 3 charge states of the insulin A-chain from representative mass spectra of **a** peak present at 7.1 min and **b** peak present at 7.4 min for a deamidated insulin sample treated with 50 mmol/L DTT and 6 mol/L urea heated at 65 °C and stirred at 300 r/min for 30 min. MS analysis was conducted in positive ion mode

The reduced deamidated insulin was also found to undergo complete disulfide cleavage, with the B- and A-chains eluting at 5.8 and 7.1 min, respectively, as was observed in the reduced native sample. However, as shown in red curve in Fig. 5, the intensity of the A-chain peak was reduced significantly, and an additional species eluted at 7.4 min. As amide groups are more polar than carboxylic acid groups, the deamidated form of the insulin chains are predicted to have a greater affinity for the reverse phase column than the native forms, and it is likely, therefore, that the peak at 7.4 min represented the deamidated A-chain. This was confirmed from the mass spectrum of the peak at 7.4 min, where a clear shift of 0.33 *m/z* in the peak cluster of the + 3 charge state can be seen relative to that of the peak at 7.1 min. This corresponded to an overall mass increase of 1 Da on the A-chain, consistent with previous findings indicating such an increase during deamidation. No evidence of deamidation was found on the B-chain, confirming that the deamidation occurred exclusively on the A-chain. Representative mass spectra showing the peak clusters for the + 3 charge states of the peaks at 7.1 min and 7.4 min of the deamidated samples are shown in Fig. 7a and b.

Subsequent to establishing the techniques described above and demonstrating their utility in characterising various known degradation products of insulin, the authors sought to determine whether these techniques could be used effectively to investigate degradation in the solid state. To do so, insulin was incubated at 60 °C/ 75% RH in the solid state for 1 week. The samples were prepared in 0.1% FA at 1 mg/mL and analysed after 1, 3, 5 and 7 d using CD, SEC and RP-HPLC.

**Fig. 8:**
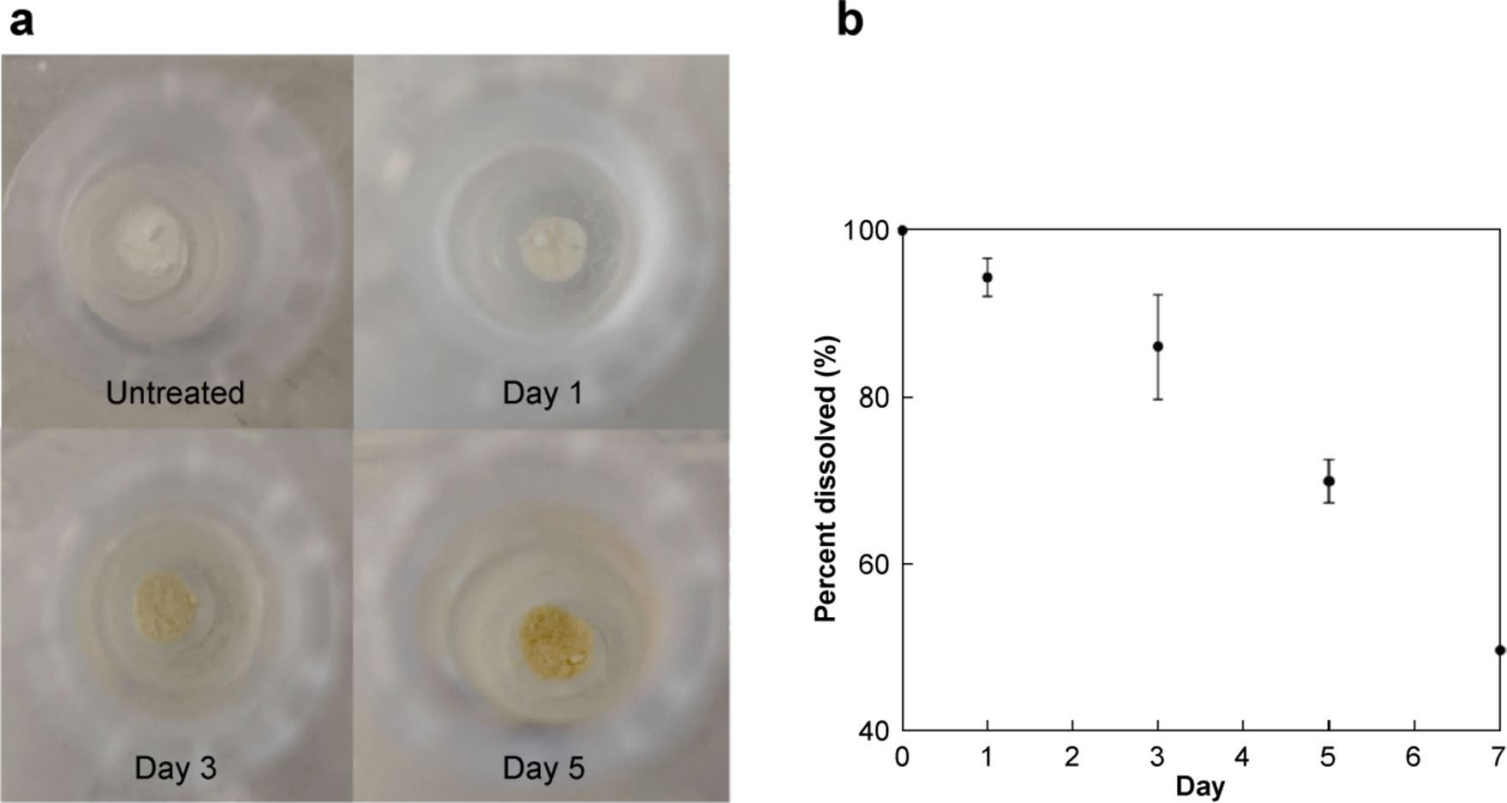
**a** Appearance of solid state insulin samples and **b** percent of insulin dissolved upon exposure to 60 °C/ 75% RH for 1, 3, 5 and 7 d, as determined by RP-HPLC (*n* = 3)

Visual inspection of the samples, shown in Fig. 8a, found that the insulin samples underwent discolouration over time, transitioning from a bright white to a dull yellow colour. This change in colour was coupled with an increase in the number of insoluble particles present in the samples over the incubation period (Fig. 8b). These physical changes to the samples served as direct evidence of degradation occurring.

**Fig. 9:**
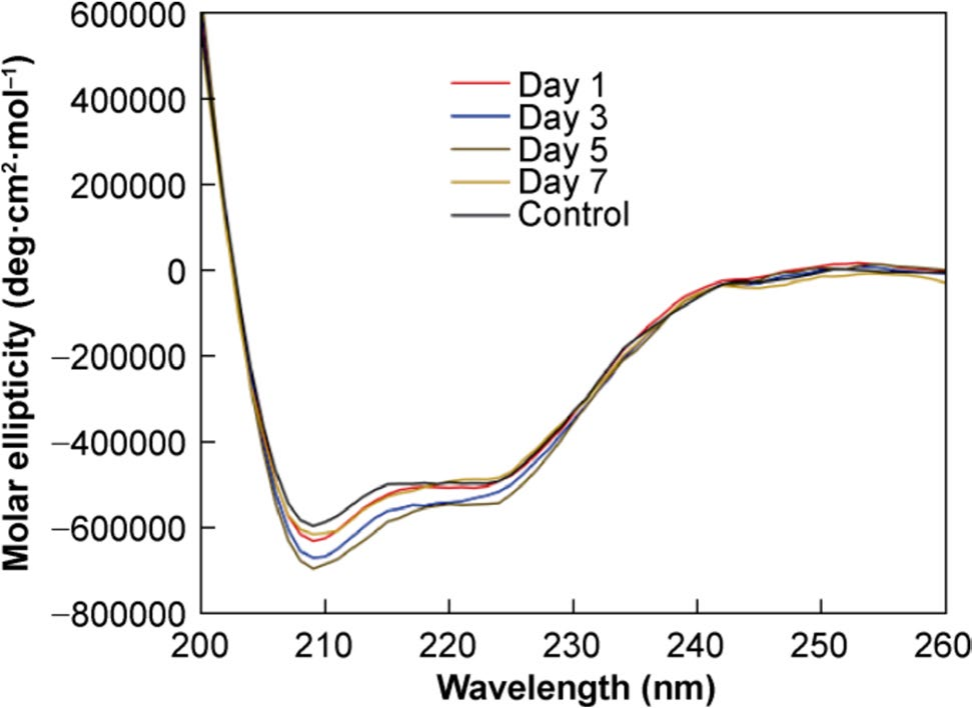
Representative far-UV CD spectra of 1 mg/mL insulin samples heated in the solid state at 60 °C/75% RH for 1 (red curve), 3 (blue curve), 5 (brown curve) and 7 d (yellow curve), and a 1 mg/mL untreated sample (black curve)

To assess the nature of the degradation, the secondary structure of the samples after reconstitution was investigated using CD spectroscopy, where representative spectra of the samples at each time point and an untreated sample are given in Fig. 9. No qualitative differences in spectral features were observed in the spectra of the samples stored at 60 °C/ 75% RH and the untreated samples, indicating any degradation that occurred in the solid state did not majorly affect the ability of the insulin molecule to fold into an ordered structure. To investigate if there were quantitative differences between the spectra, the relative intensities of the bands at 223 nm and 209 nm were determined (Table 1). All of the stressed samples were found to have a reduction in the 223 nm peak relative to native insulin in acidic conditions. Changes in the 223:209 nm ratio have been found to be related to the association state of insulin in solution, and, therefore, the formation of aggregates as a result of the stressed conditions may have resulted in a change in the hydrophobicity of the environment surrounding the helices of the stressed samples and caused a decrease in the 223 nm bands [38].

**Table 1:** Relative intensities of the 223 nm and 209 nm bands in the far-UV CD spectra of insulin samples exposed to 60 °C/ 75% RH for 1, 3, 5 and 7 d and untreated samples (*n* = 3)

Time	*I*_223 nm_:*I*_209 nm_
Day 1	0.79:1
Day 3	0.81:1
Day 5	0.79:1
Day 7	0.79:1
Untreated	0.84:1

**Fig. 10:**
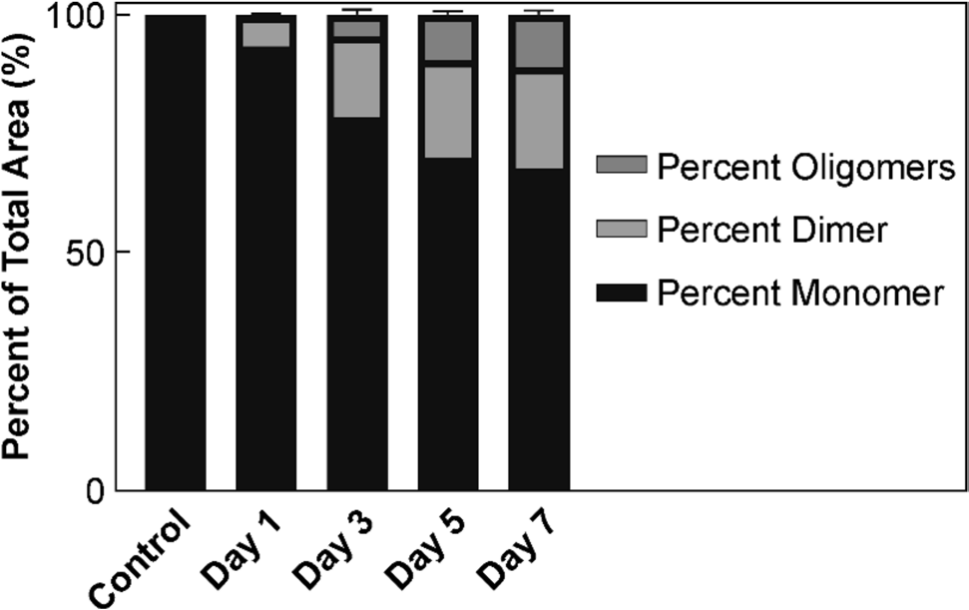
Peak areas of species present in SEC chromatogram of 1 mg/mL samples heated in the solid state at 60 °C/75% RH for 1, 3, 5 and 7 d, along with an untreated sample as a percentage of the total peak areas (*n* = 3)

To assess the aggregation profile of the samples, SEC was performed. The results of the SEC analysis are given in Fig. 10. Initially, the samples were observed to primarily form dimers, with the proportion of dimers present in the samples after Day 1 found to be 6.5%. The proportion of dimers grew rapidly between Day 1 and Day 3, composing 17% of the samples by Day 3. This initial rapid growth, however, was observed to slow, and by Day 7, dimers composed 21.1% of the samples. Larger oligomers were also found to be present by Day 3, composing 4.6% of the samples, while this increased to 11.1% by Day 7 (Fig. S4 in the supplementary information). These results, coupled with the increase in insoluble particles over time, indicated that exposure to high temperature and humidity conditions caused the samples to undergo significant aggregation over the duration of the study.

The mechanism by which insulin undergoes covalent aggregation in the solid state is complex. It has been previously reported by Strickley and Anderson that exposure to high moisture contents can lead to the formation of CIDs via a transamidation reaction [25, 26]. In the proposed mechanism, the C-terminal carboxylic acid of the Asn A21 residue undergoes intramolecular nucleophilic attack on the amide side chain to form a cyclic anhydride intermediate, which can further react with water to form an Asp A21 deamidation product or with N-terminal Gly A1 or Phe B1 residues on adjacent insulin molecules to form Asp A21-Gly A1 and Asp A21-Phe B1 covalent dimers [25]. In contrast, Constantino et al. have reported that insulin forms covalent aggregates via disulfide exchange, whereby the surface exposed A7-B7 disulfide bridge undergoes a β-elimination reaction to produce free thiols, which promote the formation of intermolecular disulfide bridges with adjacent insulin molecules [22]. However, the solubility of the samples in different reducing and non-reducing solutions was the basis for the observations made, while no discriminatory analytical techniques were used to confirm the nature of the intermolecular interactions occurring. Given that the transamidation reaction stops with the formation of CIDs, it is likely that disulfide exchange is responsible for the formation of the oligomeric species observed in this investigation, while both mechanisms may still be occurring simultaneously to form CIDs [14].

**Fig. 11:**
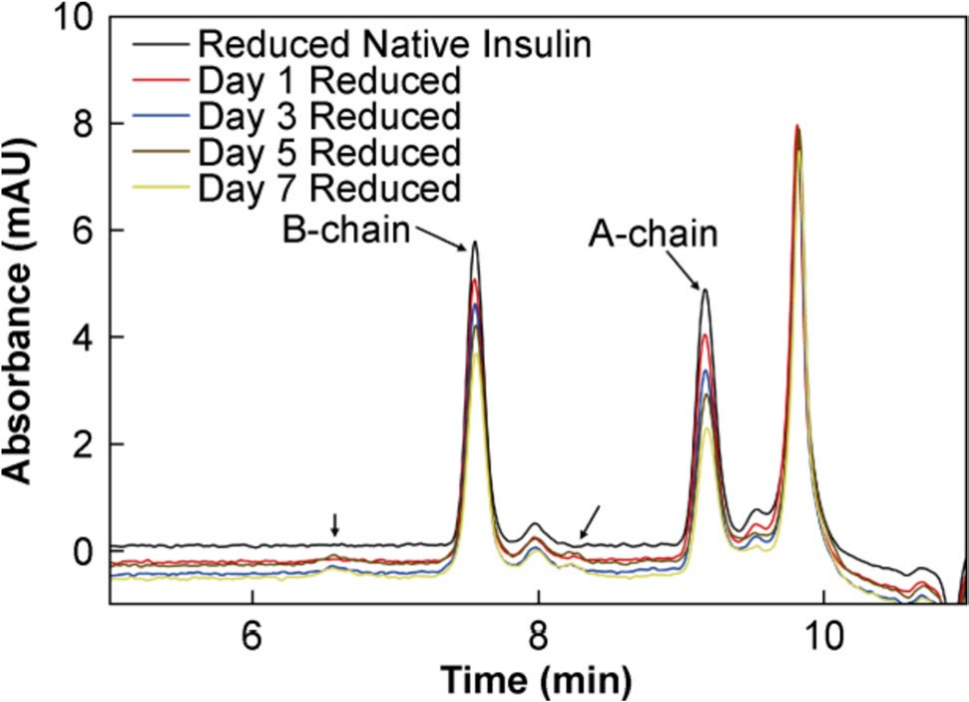
Representative SEC chromatograms of a native insulin sample (black curve) and insulin samples heated in the solid state at 60 °C/75% RH for 1 (red curve), 3 (blue curve), 5 (brown curve) and 7 (yellow curve) d, treated with 50 mmol/L DTT and 6 mol/L urea and heated at 65 °C and stirred at 300 r/min for 30 min

To investigate the mechanism of covalent aggregation, disulfide bond reduction with DTT was performed. As shown previously, reduction of the disulfide bonds in insulin with a large excess of DTT causes complete separation of the A- and B-chains. If the aggregation occurred via disulfide exchange, the aggregates should be broken down into the constituent A- and B-chains of the insulin molecules involved and will be separated by SEC. If, however, species larger than A- and B-chains are observed to elute, this is indicative of a nonreducible aggregation pathway occurring, i.e., transamidation. The SEC chromatograms of a native insulin sample and the solid state degraded samples reduced with 50 mmol/L DTT are given in Fig. 11.

From Fig. 11, minimal differences were observable between native insulin and the solid state degraded insulin on reduction with DTT. Given their presence in all samples, the peaks at 7.6 min and 9.2 min were determined to be the reduced B- and A-chains respectively due to their retention time, while the minor peaks at 7.9 min and 9.5 min were also attributed to the B- and A-chains due to their absence from all negative control samples, although the identity of these species are unknown. The peak at 9.8 min was observed to be present in all insulin and negative control samples and was attributed to the phosphate buffer salts present in the reaction buffer. There were, however, two additional peaks at very low intensity present at 6.6 min and 8.2 min in the solid state degraded samples that were absent from the reduced native insulin samples. These peaks were absent from the reduced Day 1 sample, however, were present in the reduced Day 3, Day 5, and Day 7 samples, indicating the presence of new species at very low quantities in the degraded samples on prolonged exposure to the high temperature and humidity conditions used. These peaks may represented covalently linked A- and B-chains that were unaffected by the reducing conditions and suggested that there may be a low level of transamidation occurring in the solid state degraded samples, however, there was minimal evidence for this and the predominant mechanism of covalent aggregation was via disulfide exchange.

**Fig. 12:**
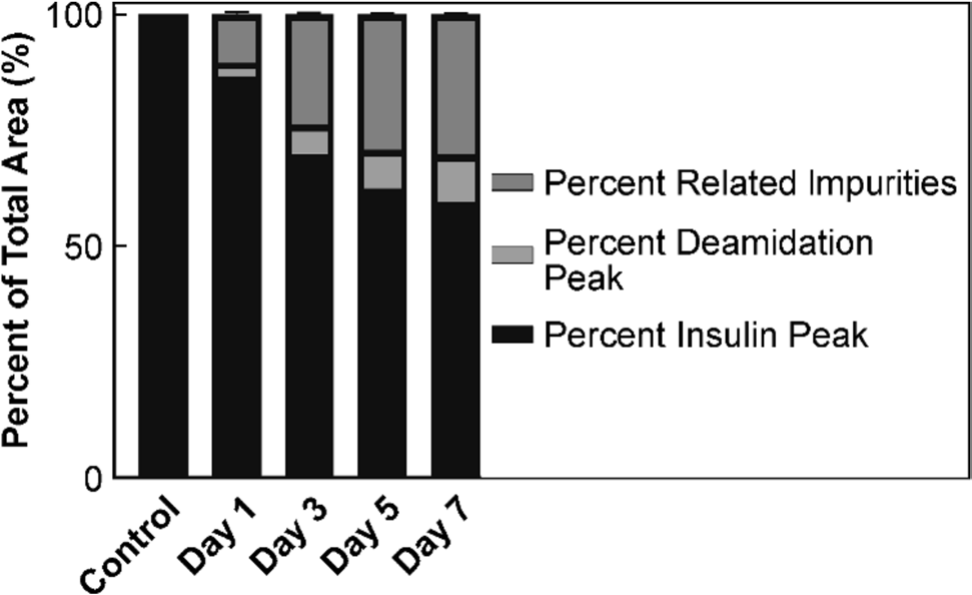
Peak areas of species present in RP-HPLC chromatogram of 1 mg/mL samples heated in the solid state at 60 °C/75% RH for 1, 3, 5 and 7 d, along with an untreated sample as a percentage of the total peak areas (*n* = 3)

Finally, RP-HPLC was performed to determine if chemical degradation was also initiated by the high temperature and humidity conditions. The results of the RP-HPLC analysis are given in Fig. 12, where it can be seen that the insulin samples underwent significant chemical degradation. Similar to the SEC results, the degradation was observed to proceed quickly and slowed between Day 5 and Day 7. As described previously, one of the most prominent chemical degradation pathways for the insulin molecule was deamidation, and this was indeed observed to occur in the solid state, making up 10.1% of the samples by Day 7.

Deamidation in the solid state is known to occur predominantly via the Asn A21 residue, as detailed above, although it has been seen to occur at the Asn B3 residue at 75% RH [39]. Deamidation was observed to be occurring on the A-chain for the solid state degraded samples, as confirmed by reductive cleavage of the insulin A- and B-chains, described previously. Representative chromatograms of a native insulin sample and the solid state degraded samples reduced with 50 mmol/L DTT are given in Fig. S5 in the supplementary information.

Multiple additional degradation peaks were observed to elute after the native insulin peak in the RP chromatograms of the solid state degraded samples, labelled Percent Related Impurities in Fig. 12 (see representative chromatogram of Day 7 sample in Fig. S7 in the supplementary information). However, LC–MS analysis did not indicate the presence of any new species between 0–1250 *m*/*z* in this region. This suggests that the additional peaks may be due to the presence of the covalent dimers and oligomers observed in the size exclusion chromatograms. Indeed, the proportion of dimers and oligomers present in SEC analysis of the samples accounts well for the proportion of the additional peaks observed in the RP-HPLC analysis. Given the large size of these species, they may have been outside of the instrument *m*/*z* range and thus were not detected.

As can be seen from the above discussion, the pathways by which insulin undergoes degradation in the solid state are similar to those observed in the solution state. Although no unfolding was observed, as expected given the reduced conformational flexibility available in the solid state, the solid state degraded samples were observed to undergo a significant amount of deamidation and covalent aggregation. Therefore, analytical techniques used for characterisation of peptides and proteins in solution can be translated to characterising degradation in the solid state, offering much higher resolution than techniques such as microscopy and thermal analysis, which are commonly used for characterising the solid state, and indeed require a much lower quantity of sample to carry out analyses.

## Conclusion

The physical and chemical stability of insulin plays an important role not only in its therapeutic activity but also its immunogenicity. As such, this investigation seeks to characterise several common degradation pathways that the insulin molecule undergoes. A short stability study was performed in which solid state insulin samples were exposed to high temperature and humidity conditions for 1 week. The results of the stability study indicated as expected, that high temperature and humidity conditions have a significant effect on the stability of the insulin molecule in the solid state. While CD spectroscopy showed that the native fold of the insulin molecule was unaffected, given the reduced conformational flexibility afforded by the solid state, the SEC and RP-HPLC analyses identified a large amount of covalent aggregation and deamidation, respectively. In particular, DTT enabled confirmation that disulfide exchange was the predominant covalent aggregation mechanism occurring in the solid state under high temperature and humidity conditions. This offers an advantage over the previous indirect approach of assessing sample solubility in different reducing and non-reducing solutions, allowing for direct detection of changes occurring on insulin’s constituent chains. These results underlined the efficacy of the techniques studied for characterising the solid state degradation pathways of insulin, offering an informative platform approach to investigating solid state degradation of peptides during oral drug product development.

### Supplementary Information

Below is the link to the electronic supplementary material.

Supplementary file1 (DOCX 2029 KB)

## Data Availability

Raw data were generated at the School of Pharmacy, University College Cork, Ireland. Derived data supporting the findings of this study are available from the corresponding author AC on request.
