# Supplementary material for: Elucidating the Degradation Pathways of Human Insulin in the Solid State
Source: J Anal Test. 2024 May 6;8(3):288–99. doi: 10.1007/s41664-024-00302-5 (PMC11338979; doi:10.1007/s41664-024-00302-5)
Supplement: Supplementary file 1 — Supplementary file1 (DOCX 2029 KB) [file 41664_2024_302_MOESM1_ESM.docx]

# Supplementary Information

# Elucidating the degradation pathways of human insulin.

# Author(s), and Corresponding Author(s)*

A. Fagan^a^, L. M. Bateman^b,c,d^, J. P. O’Shea^b^, A. M. Crean^a^*

a SSPC Centre for Pharmaceutical Research, School of Pharmacy, University College Cork, Cork, T12 YT20, Ireland.

b School of Pharmacy, University College Cork, Cork, T12 YT20, Ireland

c Analytical and Biological Chemistry Research Facility (ABCRF), University College Cork, College Road, Cork, T12 YN60, Ireland

d School of Chemistry, University College Cork, College Road, Cork, T12 YN60, Ireland

* Corresponding Author

Andrew Fagan: orcid.org/0000-0001-7375-8259; email: [andrew.fagan@umail.ucc.ie](mailto:andrew.fagan@umail.ucc.ie)

Lorraine M. Bateman: orcid.org/0000-0003-3435-861X; email: [l.bateman@ucc.ie](mailto:l.bateman@ucc.ie)

Joseph P. O’Shea: orcid.org/0000-0001-9461-8730; email: [joseph.oshea@ucc.ie](mailto:joseph.oshea@ucc.ie)

Abina M. Crean: orcid.org/0000-0001-6171-0303; email: [a.crean@ucc.ie](mailto:a.crean@ucc.ie)


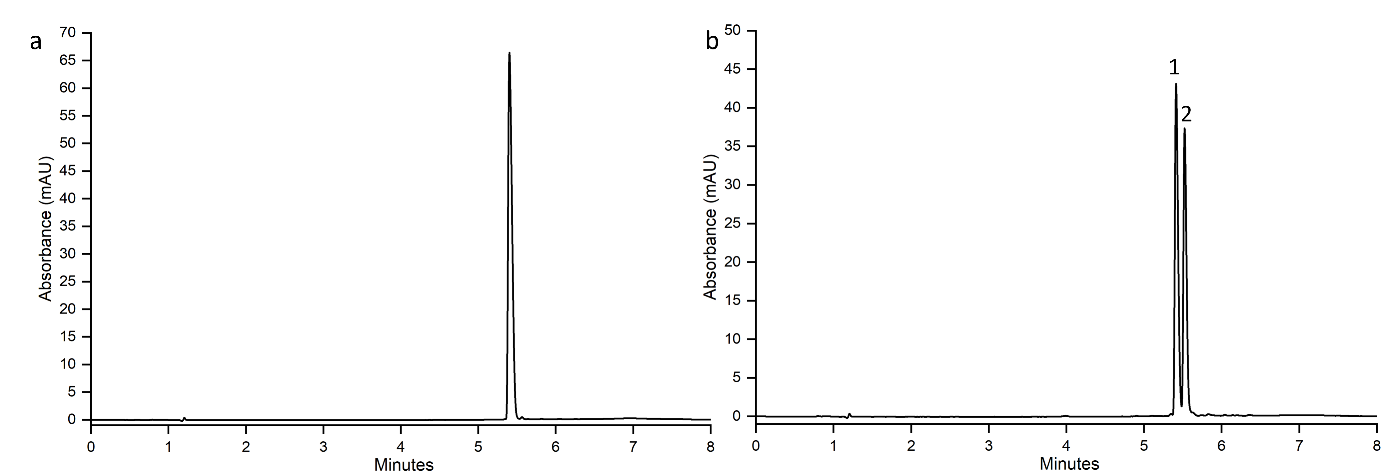


Fig. S1 Representative RP-HPLC chromatogram of (a) 1 mg/ mL insulin standard sample and (b) 1 mg/ mL insulin sample stored in 0.1% FA for 1 month, where the native insulin peak is labelled 1 and the degradant peak is labelled 2. RP-HPLC was performed using an Agilent 1260 Series HPLC system, with a Poroshell 120 SB-C18, 2.7 µm, 4.6 x 150 mm (Agilent Technologies) column. Mobile phase A (MPA) consisted of 95% H2O, 5% acetonitrile (ACN) and 0.1% formic acid (FA), while mobile phase B (MPB) consisted of 5% H2O, 95% ACN, 0.1% FA. MPB was linearly increased from an initial content of 20% to 35% over 4 min, returned to 20% MPB in 1 min and held for 3 min. The flow rate was 1 mL/ min and a total injection volume of 5 μL was used. The HPLC column was heated at 60°C. 280 nm was used as a protein specific detection wavelength


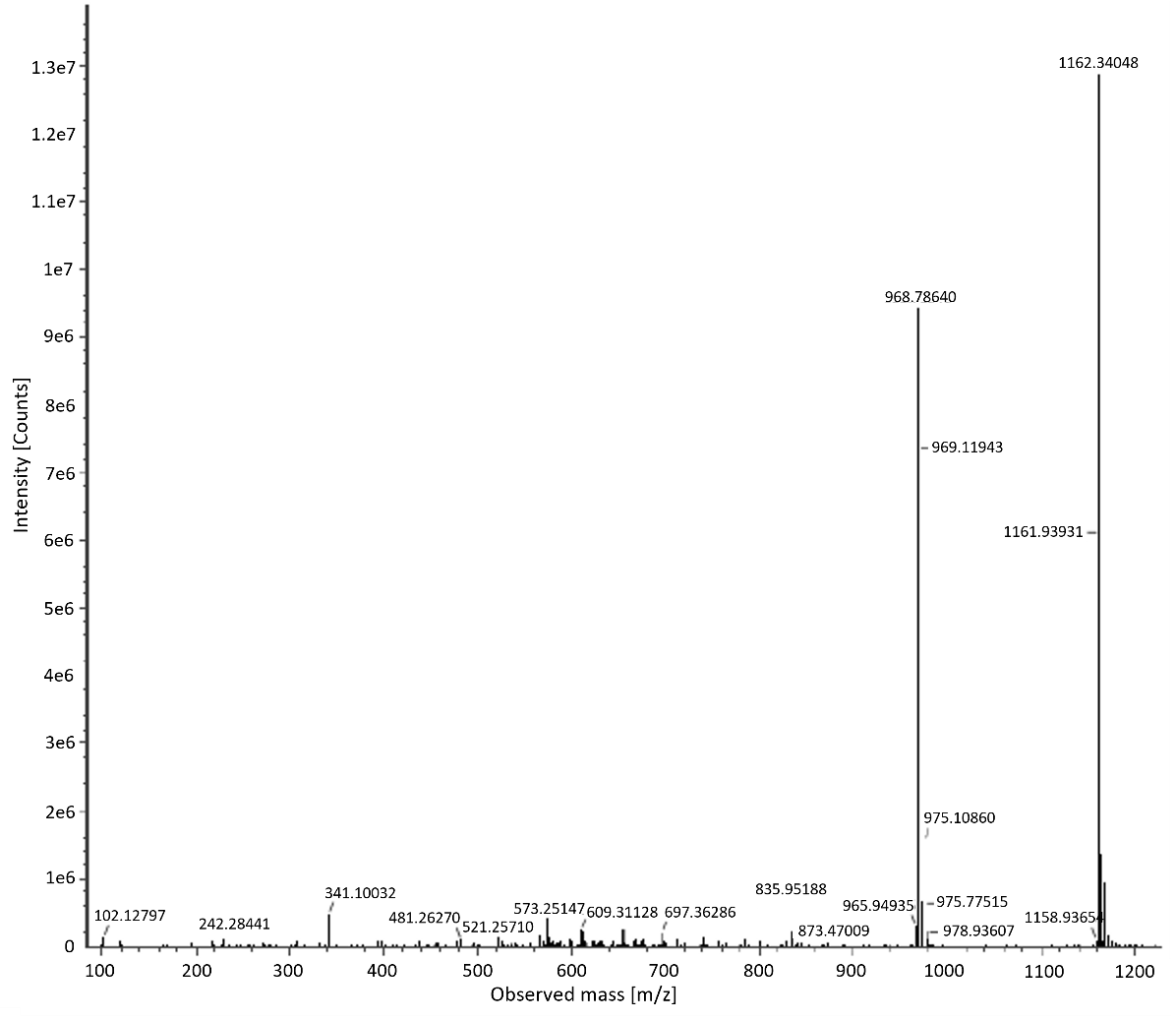


Fig. S2 Representative mass spectrum of peak 1 in Figure S1b. A Waters Acquity I-class UPLC coupled to a Vion IMS Mass Spectrometer (Waters Corporation) was used for LC-MS analysis. The chromatographic method was the same as that described in Figure S1


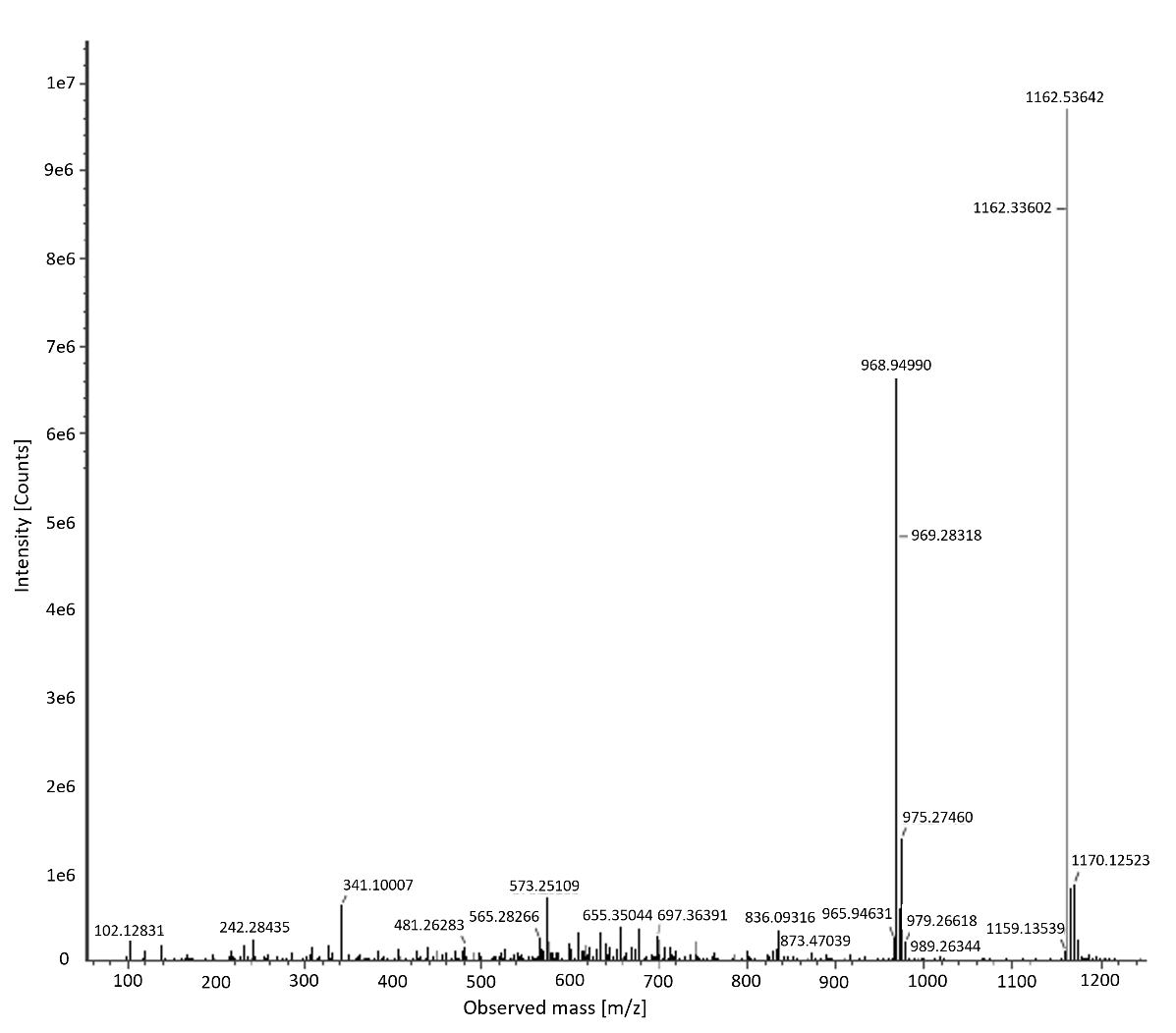


Fig. S3 Representative mass spectrum of peak 2 in Figure S1b. A Waters Acquity I-class UPLC coupled to a Vion IMS Mass Spectrometer (Waters Corporation) was used for LC-MS analysis. The chromatographic method was the same as that described in Figure S1

Table S1 Predominant ions present in mass spectra of 1 mg/ mL insulin deamidated samples, n = 3

| Sample | Species | Ion (m/z) | Charge | Mass (Da) |
| --- | --- | --- | --- | --- |
| Rep 1 | Native insulin | 968.79 | +6 | 5812.7 |
|  |  | 1162.34 | +5 | 5811.7 |
|  | Degradant | 968.95 | +6 | 5813.7 |
|  |  | 1162.54 | +5 | 5812.7 |
| Rep 2 | Native insulin | 968.79 | +6 | 5812.7 |
|  |  | 1162.34 | +5 | 5811.7 |
|  | Degradant | 968.95 | +6 | 5813.7 |
|  |  | 1162.54 | +5 | 5812.7 |
| Rep 3 | Native insulin | 968.79 | +6 | 5812.7 |
|  |  | 1162.34 | +5 | 5811.7 |
|  | Degradant | 968.95 | +6 | 5813.7 |
|  |  | 1162.54 | +5 | 5812.7 |


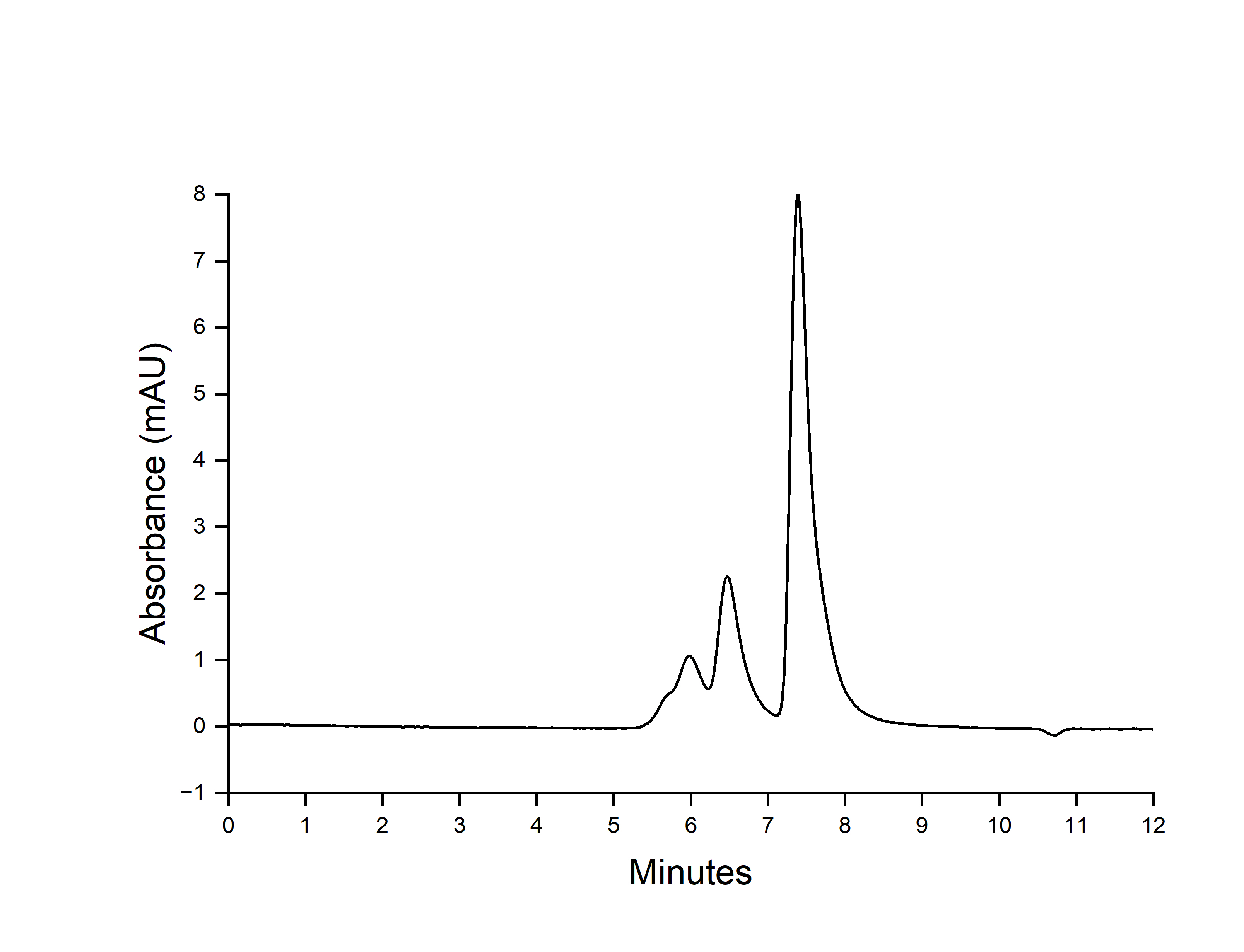


Fig. S4 Representative SEC chromatogram of 1 mg/ mL insulin sample heated in the solid state at 60°C/ 75% relative humidity (RH) for 7 days. SEC was performed using an Agilent 1260 Series HPLC system with an AdvanceBio SEC 130Å, 2.7 μm, 7.8 x 300 mm (Agilent Technologies) column. The mobile phase consisted of 65% L-arginine (1 g/ L)/ 20% acetonitrile (ACN)/ 15% acetic acid (AA). An isocratic elution method was used, with a total run time of 12 min required. A flow rate of 1.000 mL/ min and injection volume of 5 μL were used. 280 nm was used as a protein specific detection wavelength


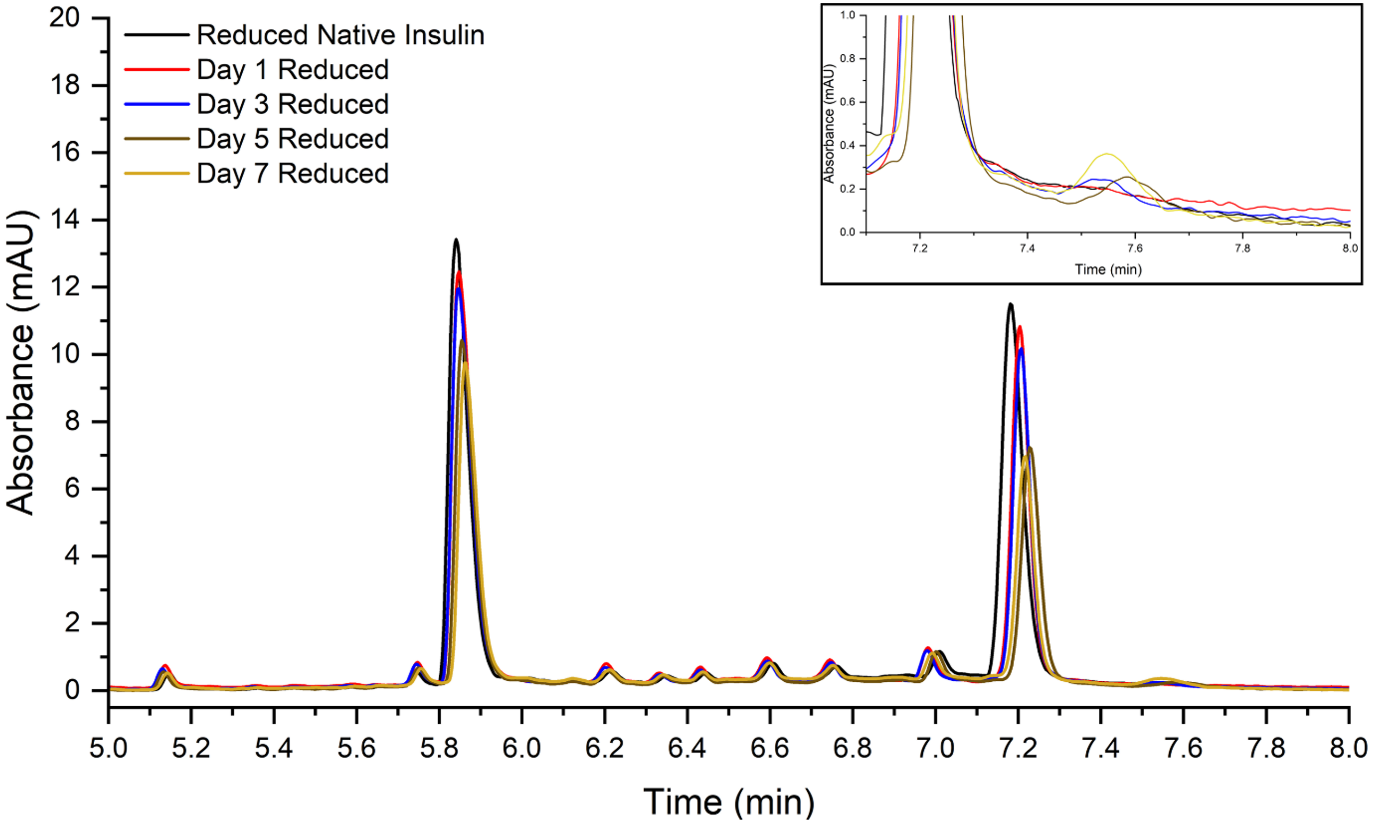


Fig. S5 Representative RP chromatograms of a native insulin sample (black) and insulin samples heated in the solid state at 60°C at 75% RH for 1 (red), 3 (blue), 5 (green) and 7 (yellow) days, treated with 50 mM DTT and 6M urea and heated at 65°C and stirred at 300 rpm for 30 min. The inset shows an expansion of the deamidation peak at ~ 7.5 min. The chromatographic method was the same as that described in Figure S1
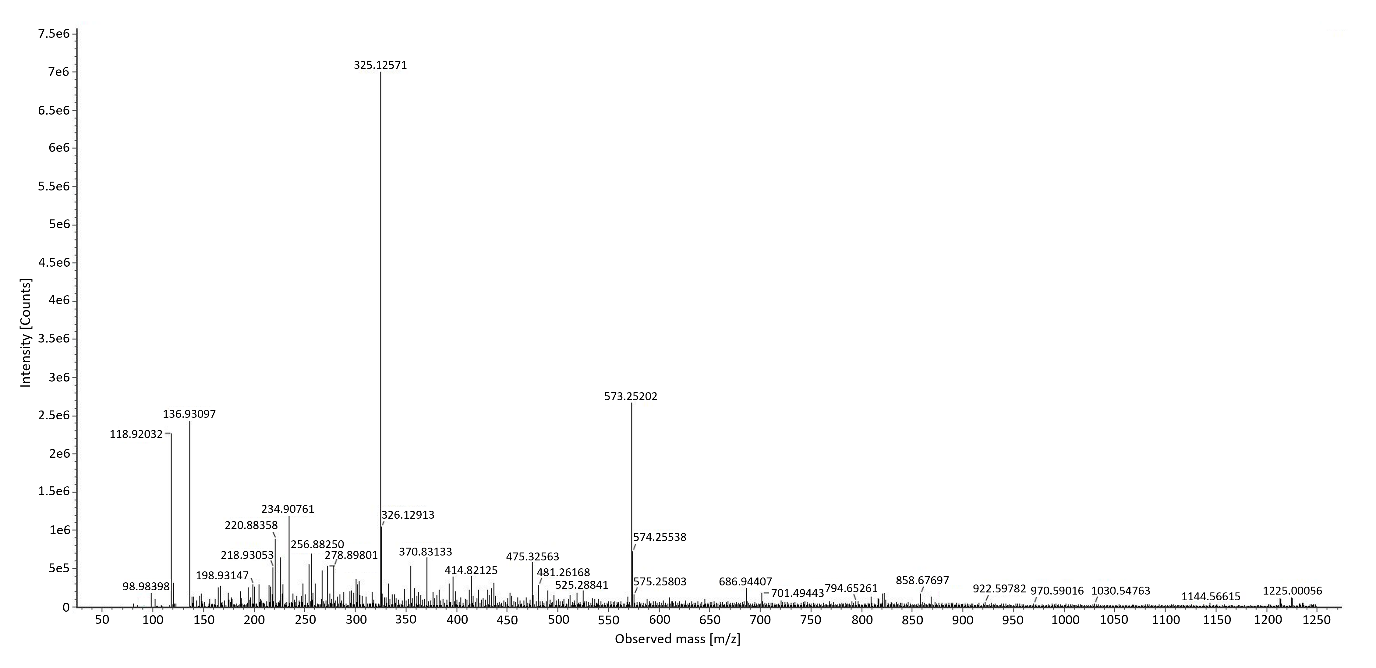


Fig S6 Representative mass spectrum showing the ions present in the region between the peaks present at 5.8 min and 7.2 min in Figure S5. A Waters Acquity I-class UPLC coupled to a Vion IMS Mass Spectrometer (Waters Corporation) was used for LC-MS analysis. The chromatographic method was the same as that described in Figure S1


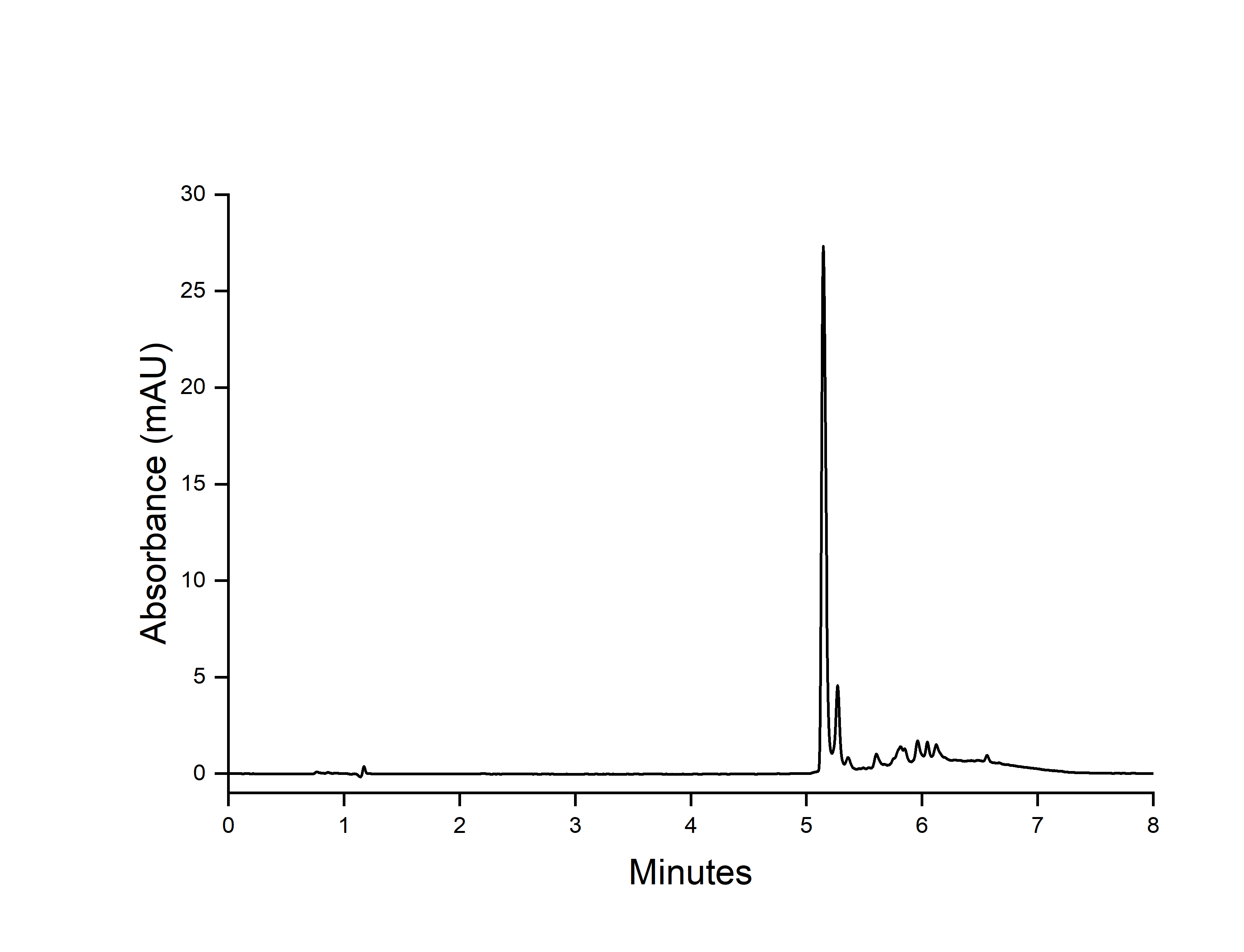


Fig. S7 Representative RP-HPLC chromatogram of 1 mg/ mL insulin sample heated in the solid state at 60°C/ 75% relative humidity (RH) for 7 days. The chromatographic system and method described in Figure S1 was used for the analysis.
